# Using host receptor as a decoy to treat COVID‐19: a solution for immune escape?

**DOI:** 10.15252/emmm.202216818

**Published:** 2022-10-18

**Authors:** Kuo‐Yen Huang, Ming‐Shiu Lin, Pan‐Chyr Yang

**Affiliations:** ^1^ YongLin Institute of Health National Taiwan University Taipei Taiwan; ^2^ Department of Clinical Laboratory Sciences and Medical Biotechnology National Taiwan University College of Medicine Taipei Taiwan; ^3^ Institute of Biomedical Sciences Academia Sinica Taipei Taiwan; ^4^ Department of Internal Medicine National Taiwan University College of Medicine Taipei Taiwan

## Abstract

There is an unmet clinical need to end the COVID‐19 pandemic. In the past 2 years, the SARS‐CoV‐2 continued to evolve and poses a critical challenge to the efficacy of the vaccine and neutralizing antibody therapies. The fifth wave of the pandemic is driven by the Omicron variants, due to their ability to evade prior immunity and their resistance to therapeutic antibodies. The report by Zhang *et al* in the current issue of *EMBO Molecular Medicine* shows that the engineered decoy ACE2 can reduce lung injury and improve survival in K18‐hACE2 transgenic mice inoculated with a lethal dose of the SARS‐CoV‐2 and potentially targets the Omicron variant.

## Graphical abstract

PC. Yang, MS. Lin, and KY. Huang discuss how engineered soluble ACE2 can be used against SARS‐CoV‐2 infection as reported by E. Procko and colleagues, in this issue of EMBO Mol Med.

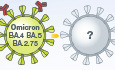

On 26 November 2021, the variant B.1.1.529 of severe acute respiratory syndrome coronavirus 2 (SARS‐CoV‐2) was defined by the World Health Organization (WHO) as a variant of concern (VOC), named Omicron. This variant is highly contagious and less virulent compared with previous strains, such as wild‐type, delta, or alpha variants. The evolution of the Omicron variant is fast and subvariants emerged (BA.4, BA.5, and BA.2.75), which are driving recent waves of increasing cases (Callaway, 2022). The accumulated mutations on the Spike protein enable the Omicron variant to evade *immunity* from *vaccines* and/or natural infections as well as the treatment with therapeutic monoclonal antibodies, such as LY‐CoV555 and Vir S309 (Dejnirattisai *et al*, 2022; Nutalai *et al*, 2022). Despite many mutations acquired on its receptor‐binding domain (RBD) of Spike, accumulating evidence implicate that Omicron Spike still binds to its host receptor, the angiotensin I–converting enzyme 2 (ACE2) (Nutalai *et al*, 2022). Previous studies have shown that soluble ACE2 or engineered decoy ACE2 fusion peptide effectively blocks SARS‐CoV‐2 infection *in vitro* and *in vivo* (Fig 1) (Monteil *et al*, 2020; Higuchi *et al*, 2021; Huang *et al*, 2021; Tsai *et al*, 2022). Indeed, a clinical trial has been done to evaluate whether the soluble ACE2 (Recombinant ACE2 only, no IgG or other peptide fusion, NCT04335136) may serve as a new drug to treat COVID‐19 infection. Unexpectedly, this wild‐type, soluble ACE2 could not prolong the survival of COVID‐19 patients. These unsuccessful results may be due to the short half‐life of soluble ACE2 and unstable storage at room temperature, which can be fixed by engineering ACE2‐tag fusion (Maiti, 2022).

**Figure 1:**
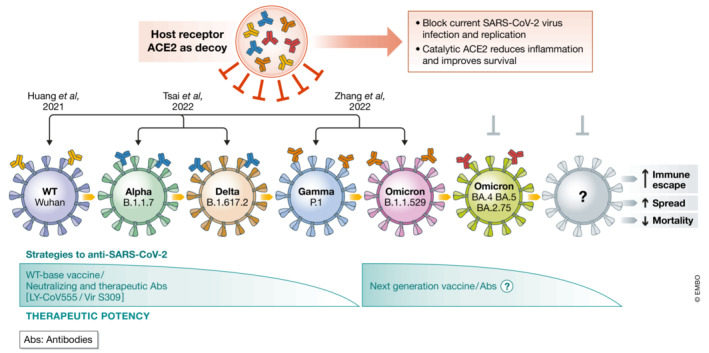
Decoy ACE2 blocks SARS‐CoV‐2 infection and replication Schematic diagram of the increasing ability of the virus to evade the immune response. The ACE2 is the critical receptor for virus binding and entry into the host cells. According to the accumulating evidence, the decoy sACE2 can effectively compete with the host ACE2 for SARS‐CoV‐2 binding and impair virus replication. [Colour figure can be viewed at wileyonlinelibrary.com]

The work by Zhang *et al* (2022) explored the use of an engineered decoy receptor fused with IgG1 (sACE2_2_.v2.4‐IgG1, with mutations T27Y, L79T, and N330Y in human ACE2) through inhalation or intravenous infusion to impair SARS‐CoV‐2 replication *in vitro* and *in vivo*. Zhang and colleagues proved that sACE2_2_.v2.4‐IgG1 impairs virus replication, recovers the body weight, and prolongs the survival rate and determined the lethal virus load (1 × 10^6^ PFU) in K18‐hACE2 transgenic mice. This group has demonstrated that sACE2_2_.v2.4‐IgG1 binding affinity to Spike protein is nearly 30‐fold higher compared with the wild‐type protein. High transmissibility and lower virulence may imply that Omicron uses a different pathway to enter host cells. However, the tight binding of the decoy ACE2 to Omicron Spike shown in this study suggests that virus evolution did not alter the entry‐rout via ACE2. This tight binding is in the range of the monoclonal antibody‐binding affinity and might be required for reaching clinical applications.

SARS‐CoV‐2 binds to ACE2 and hijacks its enzymatic activity. Zhang *et al* (2022) determined that catalytically dead or active ACE2 proteins exhibit the same RBD‐binding ability. However, this study showed the catalytic activity of the decoy ACE2 prolonged survival of SARS‐CoV‐2‐infected K18‐hACE2 transgenic mice treated by inhalation in the gamma variant model or by intravenous injection in wild‐type variant model. The previous study showed that the concentration of soluble ACE2 in serum was lower in the patients' group with viral persistence 10 days after diagnosis of infection compared with the healthy and short virus shedder group (Osman *et al*, 2021). Therefore, refilling functional ACE2 to patients may help to restore the renin–angiotensin system homeostasis to alleviate lung injury. In addition, the previous work showed that soluble ACE2 improves the effect of remdesivir in SARS‐CoV‐2 infection (Monteil *et al*, 2021). This strategy could widen the therapeutic window of the differential target drugs at the subtoxic concentration.

Although the sACE2 decoy seems to be a potential candidate for treating COVID‐19, the timeline of treatment is still unclear. Also, further studies are needed to determine the role of the catalytic activity of ACE2 in SARS‐CoV‐2 infection treatment. Considering the high mortality rate in the elderly, high‐speed virus replication, and unknown virus mutants in the future, we need more detailed information to determine the dose of sACE2 and treatment duration of SARS‐CoV‐2 infection.

## Disclosure and competing interests statement

The authors declare that they have no conflict of interest.
